# Nasolabial dirofilariasis in humans: a case report

**DOI:** 10.1007/s10006-025-01469-6

**Published:** 2025-10-23

**Authors:** Pietro Salvatori, Gisele de Rezende, Emanuela Bonoldi, Guido M. Petazzi, Maria Menini

**Affiliations:** 1 Formerly, ENT Department, Humanitas San Pio X Hospital, Milan, Italy; 2 Laboratory of Anatomic Histopathology and Cytogenetics, Department of Haematology, Oncology and Molecular Medicine, Niguarda Cancer Centre, Milan, Italy; 3 Division of Prosthodontics, Department of Surgical Sciences (DISC), University of Genoa, Genoa, Italy

**Keywords:** *Dirofilaria repens*, Subcutaneous dirofilariosis, Zoonosis, Maxillofacial region, Oral pathology, Mosquito, Facial swelling

## Abstract

We report a case of dirofilariasis affecting the nasolabial region, which appears to be only the second case described in the worldwide medical literature. The patient’s medical history, diagnostic work-up, and treatment are described, with particular emphasis on aspects relevant to dental practitioners.

## Introduction

Dirofilaria species are parasites of dogs, cats, and wild carnivores. The two most common species are *D. repens* and *D. immitis*. *D. repens* resides in the subcutaneous tissues of its natural hosts, sometimes for prolonged periods. Microfilariae (first-stage larvae) may enter the peripheral bloodstream, where mosquitoes ingest them, becoming the intermediate hosts. Infected mosquitoes transmit third-stage (L3) larvae to new hosts during blood meals [1].

Humans occasionally serve as accidental hosts. *D. repens* typically causes subcutaneous or ocular infections, whereas *D. immitis* is more often associated with pulmonary disease [2]. Bytyqi et al. [3] reported that most published oral–facial filarial infections are due to *D. repens*. Other oral–facial localisations have also been documented [4–7].

Here, we present a rare case of nasolabial dirofilariasis, representing only the second such case reported in the international literature.

## Case presentation

A 45-year-old woman presented with a subcutaneous nodule on the right nasolabial region. She had no relevant comorbidities.

Two and a half years before the final diagnosis, she had developed recurrent fever and conjunctivitis. A chest X-ray was unremarkable.

Six months later, she experienced pain, redness, and swelling of the right ankle, accompanied by painful nodules on the same leg. A diagnosis of erythema nodosum was made, and steroid therapy was prescribed, with a good clinical response.

A pharyngeal swab subsequently identified *Streptococcus pyogenes*, which was successfully treated with benzylpenicillin. Later, the patient developed polyarthralgia involving both temporomandibular joints.

Twenty months after symptom onset, she reported nocturnal episodes of bite-like sensations on the abdomen and thighs, followed by skin fissures with marked inflammation, induration of the surrounding tissue, and delayed healing.

She also developed a persistent non-productive cough without fever and recurrent itchy swellings of the left arm, lasting 3–4 days each and recurring for three months, despite treatment with amoxicillin–clavulanate and prednisone.

Eventually, a small subcutaneous nodule appeared on the right upper lip, which spontaneously regressed and then recurred, accompanied by angioedema. The angioedema resolved but left residual oedema at the lip–nose junction and mild numbness of the right hemiface.

**Fig. 1:**
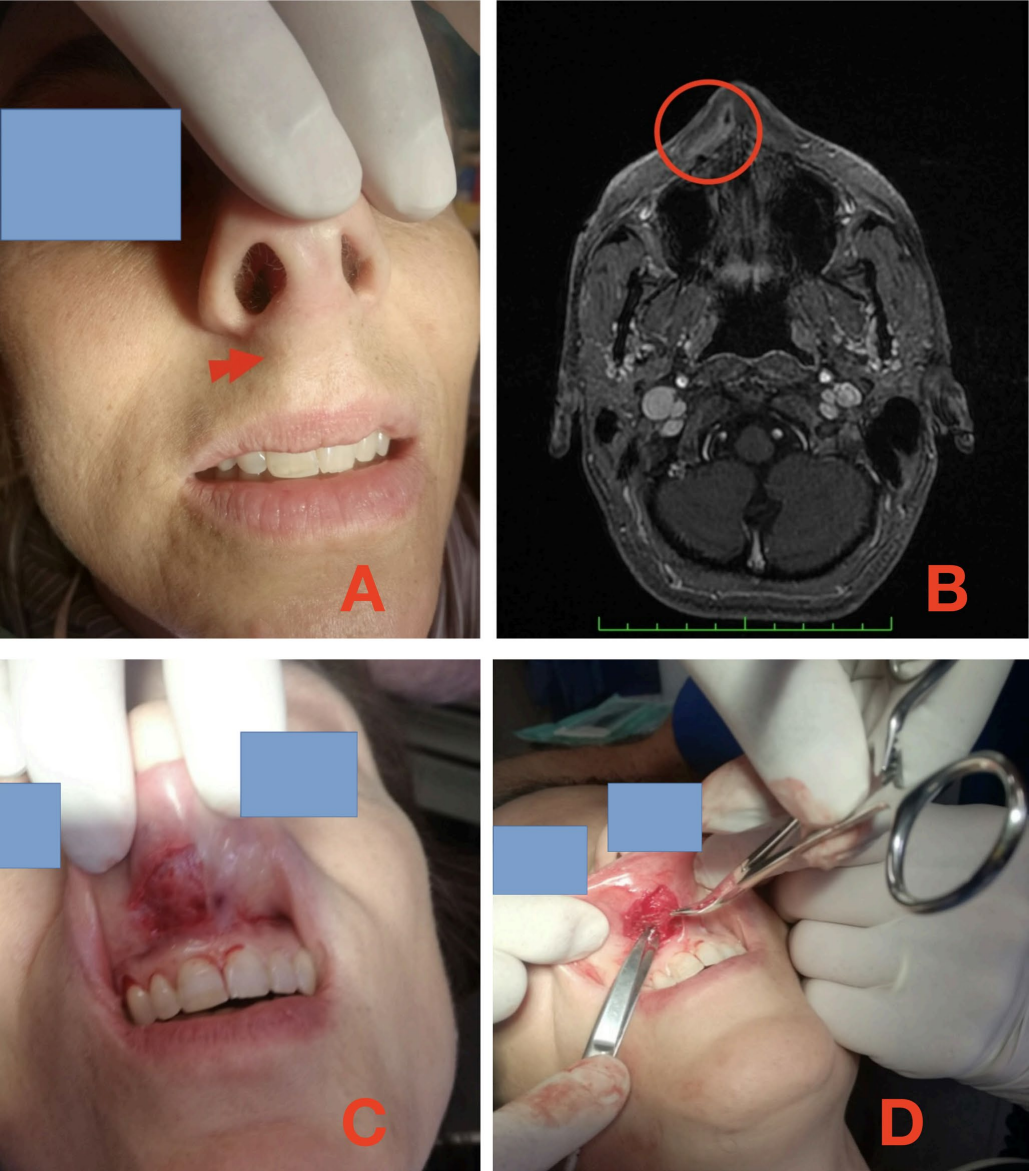
**A**: preoperative view; red arrow indicates the swelling. **B**: Nuclear Magnetic Resonance; red circle surrounds the lesion. **C**: incision. **D**: dissection

Intermittent tachycardia occurred during this period. Four months after the lesion first appeared, the upper lip again became swollen (Fig. 1A).

### Investigations

The patient’s ultrasound examination showed a small cyst containing corpuscles suspected of being parasites. The suspect was confirmed by Nuclear Magnetic Resonance (Fig. 1B).

Meanwhile, the tachycardia episodically recurred, not being well controlled with β-blockers.

A Cone-Beam CT scan did not show any lesion within the upper incisal bone.

### Treatment

After 7 months from the nodule’s appearance, the patient underwent its excision under local anaesthesia. To avoid a labial scar, the vestibular approach was chosen. After mucosal incision (Fig. 1C), the nodule was identified and carefully dissected through the muscular layer, up to the right side of the anterior nasal spine (Fig. 1D). Care was taken to leave an adequate bulk of normal tissue surrounding it. Primary closure was achieved in layers.

### Histopathological findings

**Fig. 2:**
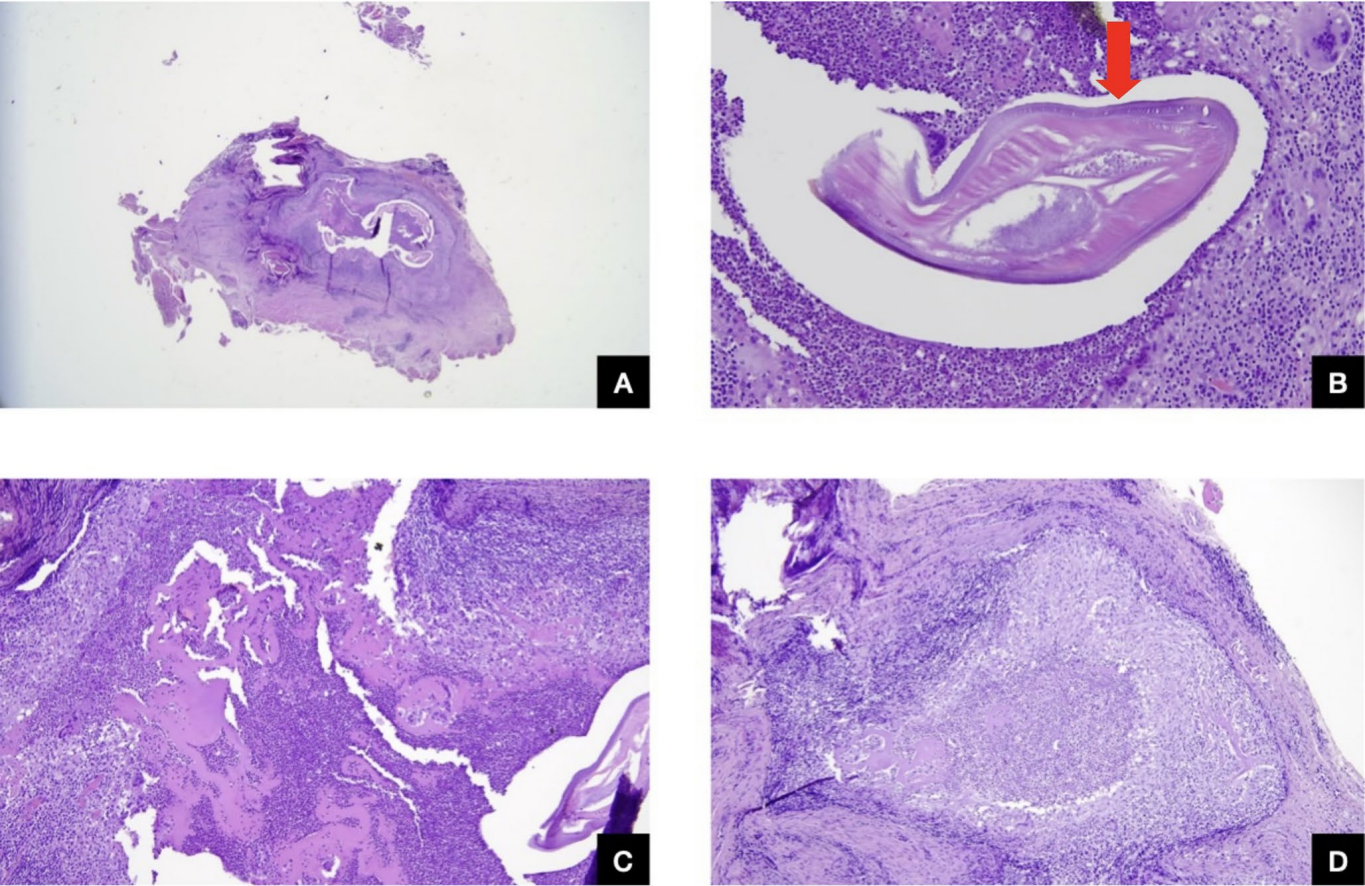
**A**: Degenerating filaria (overview). H&E stain, 25x magnification. **B**: Detail view. Degenerating filaria. Note the chitinous cuticle (arrow) with longitudinal ridges and prominent lateral cords (smooth muscle fibres). H&E stain, 400x magnification. **C**: Inflammatory infiltrate composed of lymphocytes, plasma cells, histiocytes, and eosinophils. H&E stain, 200x magnification. **D**: Abscess cavity with granulomatous component and multinucleated giant cells surrounding the worm. H&E stain, 200x magnification

Histopathology revealed connective tissue with adherent muscle showing chronic active inflammation, abscesses, abundant eosinophils, and multinucleated giant cells. At the centre of the inflammatory response were degenerated cylindrical structures consistent with nematode remains, highly suggestive of Dirofilaria (Fig. 2A–D).

Definitive identification of *D. repens* was confirmed at a reference laboratory.

### Outcome

**Fig. 3:**
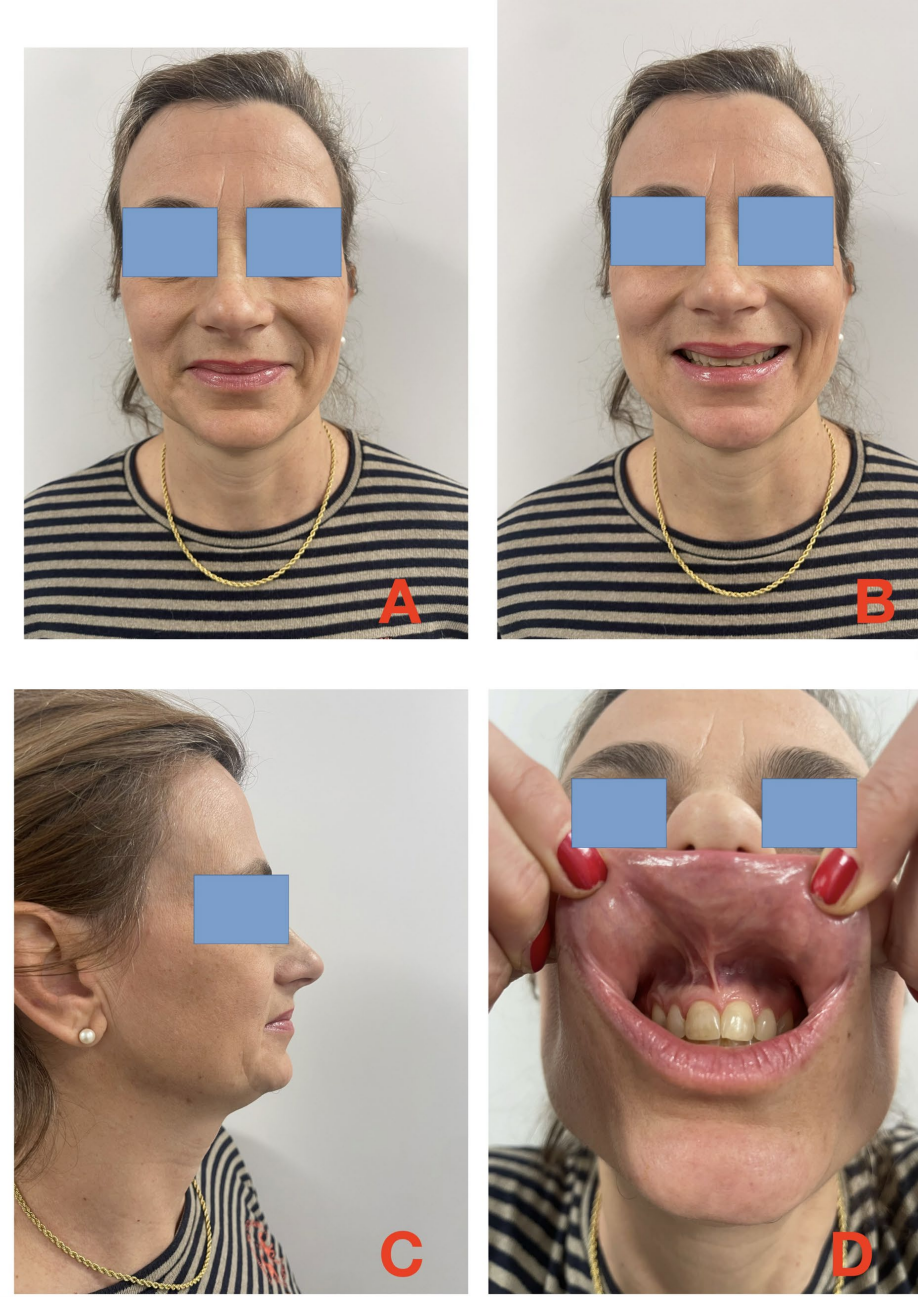
4-year outcome **A**: static, **B**: smiling, **C**: side view, **D**: mucosal plan

The postoperative course was uneventful. The patient healed normally and required no further treatment. She remains in good health four years after surgery (Fig. 3A–D).

## Discussion

Bytyqi et al. [3] conducted a comprehensive review of oro-facial subcutaneous dirofilariasis, a disease caused by Dirofilaria species (*D. repens* and *D. tenuis*), for which domestic dogs and wild canids serve as definitive hosts. During a blood meal taken from an infected canid, infective larvae are ingested by mosquito species (e.g. Aedes spp., Anopheles spp., or Mansonia spp.), which ultimately renders them infectious [8, 9]. During a subsequent blood meal taken from humans, infective dirofilarial larvae are released into the human accidental host, in whom the larvae eventually develop into adults, migrating in the subcutaneous tissue or forming granulomatous nodules. If manifesting in the oro-facial region, subcutaneous dirofilariasis could be etiologically important in explaining oro-dental clinical phenomena, such as swellings, nodules and potentially even (tooth) pain, caused by edematous swelling exerted onto local nerves by a migrating adult worm, or a juxtaposed granulomatous nodule. Local pressure also inhibits blood flow, which in theory could cause dental ischemia and thereby lead to tooth loss. Lastly, it is theoretically possible that systemic or local inflammatory activity caused by filarial infection creates a procarcinogenic environment, potentially contributing to the formation of oral cancer.

Dirofilariasis was most commonly reported from the WHO European Region (53.9%; 55/102), and the WHO South-East Asian Region (37.3%; 38/102) followed by a few reports from the WHO Region of the Americas (4.9%; 5/102), the WHO Eastern Mediterranean Region (2.9%; 3/102) and the WHO Western Pacific Region (1%; 1/102) [3].

The median age of dirofilariasis cases was 39 years (IQR: 28 to 52 years), and females and males were equally often affected (49% [49/101] and 51% [52/101], respectively). Oro-facial dirofilariasis was almost exclusively caused by *D. repens* (98%; 81/83), with only two cases attributed to *D. tenuis* (2%). However, it is worth mentioning that definitive Dirofilaria species diagnosis was not performed in 18.6% of cases (19/102). The most commonly affected oro-facial site was the cheek (63.7%; 65/102), followed by the face (17.7%; 18/102), the lip (7.8%; 8/102) and the jaw (6.9%; 7/102); furthermore, there was each a case with involvement of the nasolabial region, the oral cavity, the soft palate and the tongue, respectively. Cases for whom therapy was reported (*n* = 55) mainly underwent surgical removal (89%; 49/55) or surgical removal including anti-filarial treatment (5%; 3/55). In two cases, the swelling was squeezed, and a worm was recovered, and lastly, in one case, the nodule developed into an abscess, which burst and revealed a white worm. They also stated that oro-facial dirofilariasis caused by *D. repens* represents the vast majority of oro-facial filariasis, followed by a small non-dirofilarial minority represented by *W. bancrofti* and *O. volvulus* [3]. 

Oro-facial dirofilariasis manifests exclusively as nodules or swellings in different tissue locations. This is in line with the biology of the adult stages of *D. repens* and *D. tenuis*, both of which are commonly found in subcutaneous tissue [8]. While swellings (some of which can appear to migrate) are often described as being caused by the active migration of the adult worm, subcutaneous nodules are formed by adult worms whose migration is rendered stationary by the host’s immune system [10]. Characteristically, many case reports reported swellings of subcutaneous tissue for weeks to months, which often spontaneously increased and decreased in size and finally manifested into a single, mobile, soft, or firm nodule. Also, in line with the literature, these nodules and swellings are mostly asymptomatic; however, they can become painful if the immune system of the host attempts to clear the active infection [11]; again, this is congruent with our findings since pain and dysesthesia combined constituted 71% (10/14) of symptoms in symptomatic oro-facial dirofilariasis. Interestingly, most manifestations of oro-facial dirofilariasis occurred on the cheek and face, and to a lesser extent, on the lip, jaw, and other areas. However, it is believed that these are not true predilection sites, but artefacts resulting from categorising the human body into anatomical regions, some of which are naturally larger than others. Furthermore, the recommended treatment of general dirofilariasis caused by *D. repens* or *D. tenuis* is surgical removal [11].

The vast majority of dirofilariasis was caused by *D. repens* (98%), followed by *D. tenuis*, while *D. immitis* caused no cases. This phenomenon can be explained via parasite biology since adults of *D. immitis* are described to inhabit the intravascular space primarily of pulmonary arteries, and not subcutaneous tissue (which is the case for adults of *D. repens* and *D. tenuis*) [8].

Deducting from the reported extensive review [3], the case we report should be the second in the world to affect the nasolabial region and falls within the 20% of symptomatic cases. Pre-diagnosis course fully replied to the most common evolution: lump migration, sensorial impairment of the face, immune system reaction aimed to eliminate the pathology, months from the beginning of the symptoms/signs to the removal.

Parasites can rarely be identified at histopathological examination, and, most often, only degenerated, dead nematode structures are observed. This finding can sometimes be the only way to diagnose such infections. The patient’s referred history of exposure can also provide supportive information.

In our case, we examined histological sections of adipose and skeletal muscle tissue from the nasolabial area. We observed a prominent inflammatory infiltrate consisting of lymphocytes, plasma cells, histiocytes, eosinophils, and giant cells. Additionally, a central area of suppuration with abundant neutrophils surrounding a degenerating filaria was evident. We were able to identify a thick, laminated chitinous cuticle with longitudinal ridges and large lateral cords, displaying the characteristic arrangement of smooth muscle fibres. The presence of these structures was crucial in suggesting a diagnosis of Dirofilaria sp.

It is crucial to consider that many different worms share morphological features; moreover, other types of foreign bodies should be considered in the differential diagnosis, together with infections, tumors, inflammatory dyseases and cysts. This point highlights the importance of thorough examination and evaluation of all possible factors when diagnosing parasitic infections.

Molecular methods, such as PCR, can detect parasite DNA in blood and may be helpful in occult or suspected cases [12]. However, in this patient, histology alone was sufficient to establish the diagnosis.

Meanwhile, in the absence of adequate clinical and epidemiological research results, oral health providers should consider parasitological etiologies in the manifestations of oro-facial diseases, particularly in patients at high risk of carrying a specific parasitic infection. Unfortunately, oral health providers were often not exposed to the idea in dental medicine curricula that parasites can be an underlying cause of certain oro-facial diseases. This educational gap is even further complicated by the fact that parasite-specific characteristics (e.g. life cycles, risk factors, or disease manifestation) can be vastly different among parasitic diseases.

## Data Availability

No datasets were generated or analysed during the current study.
